# Etiology of white pupillary reflex in pediatric age group

**DOI:** 10.22336/rjo.2022.8

**Published:** 2022

**Authors:** Muhammad Israr, Khalil Khan Zahir, Adnan Khattak, Irfan Ullah Khattak, Nazli Gul

**Affiliations:** * Fellow Pediatric Ophthalmology HMC Peshawar, Pakistan; ** Health Department KPK, Pakistan; *** HMC, Peshawar, Pakistan; **** KTH Peshawar, Pakistan

**Keywords:** leukocoria, retinoblastoma, retinal detachment, coats disease, persistent fetal vasculature

## Abstract

**Background:** Leukocoria means white pupil. Normal pupil appears black in children and adults. The typical red reflex is due to retro-illumination of choroidal vessels reflected via the retina, vitreous humor, crystalline lens, aqueous humor, pupil, and cornea. If there is interference in these structures, it would result in a changed red reflex, or leukocoria. Immediate family members are highly likely to detect the first indicator and the pediatrician or general ophthalmologist is usually the first to be visited.

The aim of the study was to find out the prevalence of common causes of white pupillary reflex in children, to undertake early diagnosis and treatment, and to reduce morbidity and death. This study aimed to see how common it is for children to have a white pupillary reflex when they visit a pediatric ophthalmologist.

**Objective:** Determine the incidence of conditions that cause a white pupillary reflex in children who visited Hayatabad Medical Complex Hospital in Peshawar.

**Materials and methods:** This study was carried out in the Ophthalmology unit of HMC Hospital Peshawar, from January 2021 to December 2021. 168 patients were enrolled in the study. We included all patients of up to 10 years and both genders with the above findings. Workup for leukocoria was done to find the exact cause that included fundoscopy, B-Scan, MRI, and CT scans. Examination under anesthesia (EUA) was carried out for uncooperative children for detailed fundus examination. Patient data was recorded and a proforma was made to collect all the necessary information. Family history was taken in detail during this study.

**Results:** The most common cause of aberrant pupillary reflex in children aged 1 to 10 years was cataract, 79.76 percent of patients having it. Retinoblastoma (12.5%), Coats disease (3.5%), retinal detachment (2.9%) and persistent hyperplastic vitreous (PHPV) (1.1%) were other notable causes found.

**Conclusion:** Leukocoria is a critical clinical finding, and if parents or primary care physicians notice it, the patient requires a complete follow-up examination by a pediatric ophthalmologist to determine the etiology.

## Introduction

Leukocoria is a Greek word that means “white pupil”. In contrast to the usual red reflex, leukocoria presents as a yellowish, pale, whitish, or otherwise abnormal reflection of light in one eye or both eyes. Normal pupil color is black in young age and slightly grey in old age [**1**]. Mydriasis or photography can reveal leukocoria, which is an unusual pupillary reflex. It is frequently the first symptom of a variety of significant intraocular problems [**2**].

Cataract, retinoblastoma, retinal detachment, retinopathy of prematurity, pupillary membrane persistence, persistent hyperplastic vitreous (PHPV), endophthalmitis, optic nerve coloboma, iris heterochromia, and ametropia are common causes leading to a white pupillary reflex [**3**].

The purpose of this study was to identify the common causes leading to white pupillary reflex in our setup, and once found, ophthalmologists should perform a timely intervention to minimize morbidity and mortality.

## Materials and methods

This study was carried out in the Department of Ophthalmology of Hayatabad Medical Complex, Peshawar. The study was cross sectional, with non-probability consecutive sampling. The study duration was 1 year, from January 2021 to December 2021, after the institutional ethical committee approved the proposal. A total of 168 patients were enrolled in this study and an informed consent was obtained from parents/ guardians. Workup for leukocoria was carried out to find out the exact cause, which included fundoscopy, B-Scan, MRI, and CT scans. Examination under anesthesia was carried out in uncooperative children for detailed ocular examination and fundoscopy. The inclusion criteria were children between 1-10 years old, both genders having symptoms for more than one week being included. Children who had previous history of trauma or ocular surgery were excluded.

## Results

Patients were divided into three age groups, 75 (44.6%) in the 1-2-year-old group, 57 (33.9%) in the 3-5-year-old group, and 36 (21.4%) in the 5-10-year-old group. The study included range was from 1-10 years, with an average age of 4.27 years ± 0.80 SD (**Table 1**).

**Table 1:** Age distribution

Age in Years	Frequency	Percentage (%)	Mean ± SD
1-2	75	44.6	
3-5	57	33.9	
5-10	36	21.4	4.27 ± 0.80
Total	168	100	

**Fig. 1:**
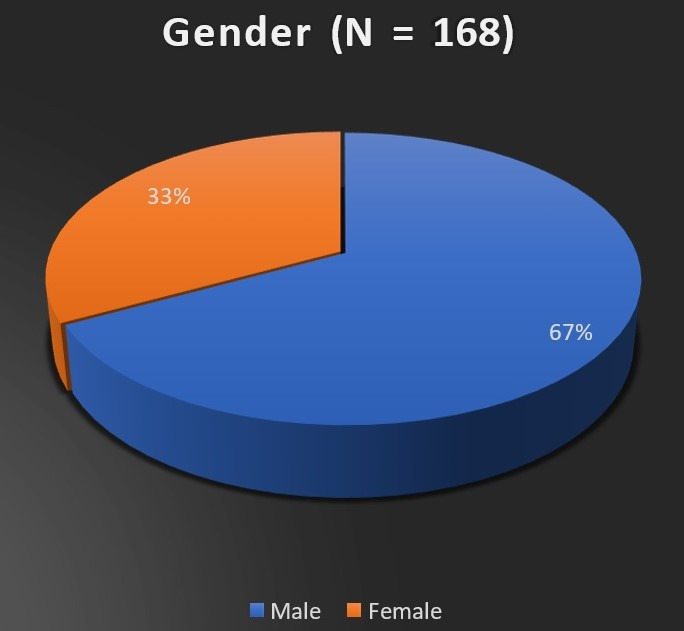
Gender distribution

Of 168 patients included in the study, 113 (67.26%) were males, and 55 (32.73 %) were females, as represented in **Fig. 1**.

**Fig. 2:**
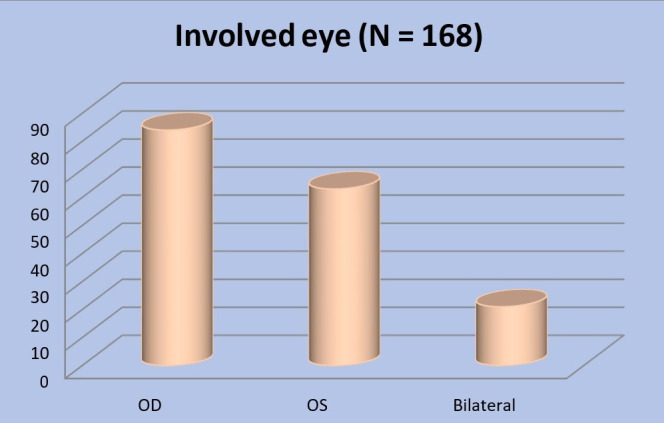
Involved eye

The data was afterwards segmented by whether a single (right/ left) or both eyes presented leukocoria. In 84 cases (50%), the right eye was affected, in 63 cases (37.5%), the left eye, and in 21 (12.5%) cases, both eyes (**Fig. 2**).

Patients who presented with a white pupillary reflex (leukocoria) were also noted for the duration of symptoms. Ninety-three patients (93), i.e., 55.35%, had symptoms for more than one week, while 75 (44.64%) had symptoms for more than two weeks. All cases were then divided into groups based on causes. Cataract was present in 134 (79.76%), followed by retinoblastoma in 21 patients (12.5). Retinal detachment 5 (2.9%), persistent fetal vasculature 2 (1.1%), and Coats disease 6 (3.5%), were also notable findings of our study (**Table 2** and **3**). 

**Table 2:** Causes of white pupillary reflex (leukocoria)

Causes	N	Percentage (%)
Cataract	134	79.76
Retinoblastoma	21	12.5
Retinal detachment	5	2.9
Persistent fetal vasculature	2	1.1
Coats disease	6	3.5
Total	168	100

**Table 3:** Causes of white pupillary reflex (leukocoria)

N/168	Cataract	Retinoblastoma	Retinal detachment	Persistent fetal vasculature	Coats disease
Causes	134	21	5	2	6

## Discussion

Leukocoria (or white pupil) presents as a yellowish, pale, whitish, or otherwise abnormal reflection of light in one or both eyes. Normal pupil color is black in young and slightly grey in old age [**1**]. The retro-illumination of normal choroidal vessels reflected via the retina, vitreous humor, crystalline lens, aqueous humor, pupil, and cornea cause the classic red reflex.

**Fig. 3:**
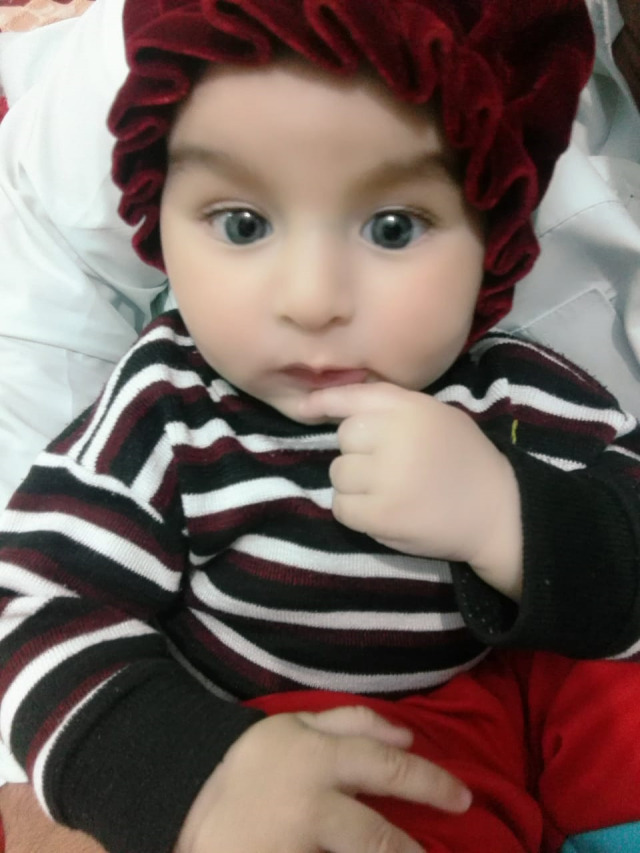
Normal pupillary reflex

**Fig. 4:**
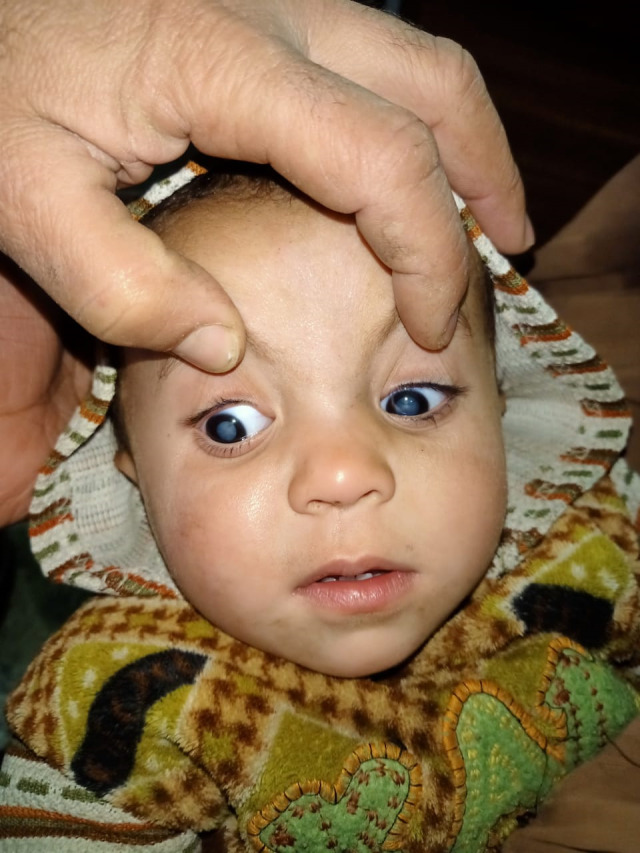
White/ abnormal pupillary reflex

Family members are more likely to detect it by chance, either through direct observation of the child or through images shot with the flash setting that show a white pupil [**4**]. A regular neonatal and pediatric examination must include testing this red pupillary reflex [**5**].

**Fig. 5:**
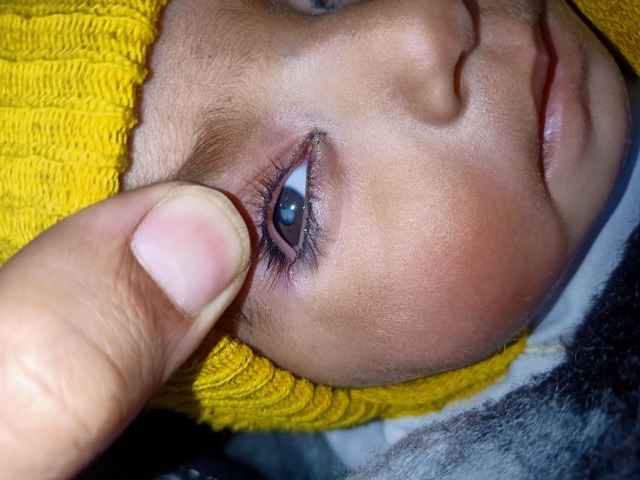
Leukocoria caused by congenital cataract in a 1-year-old child

**Fig. 6:**
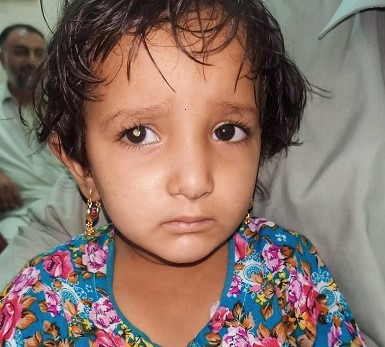
Retinoblastoma causing leukocoria in a 5-year-old female child

A whitish pupil is the initial sign of a variety of eye issues in children [**6**]. Pediatric cataract can be congenital or acquired, unilateral or bilateral [**7**], and curable in most cases (**Fig. 5**). Pediatric cataract is one of the most common causes (for 5–20% of pediatric blindness worldwide [**8**]) of blindness and severe vision impairment in children, despite its rarity [**9**]. Globally, 200 000 children are blind due to cataracts, with 20 000-40 000 children born with congenital cataract each year [**10**]. In terms of human morbidity, economic loss, and societal hardship, blindness in children, due to cataract, is a considerable concern in poor countries [**11**]. The most frequent intraocular malignancy in children and teenagers is retinoblastoma (RB) (**Fig. 6**), and it poses a significant threat to their vision and lives [**12**]. It has a 100% mortality rate if not treated in time. The precise pathophysiology of retinoblastoma is unknown [**13**].

Rizwana D et al. found that cataract was 77.3%, retinoblastoma 5.8%, retinal detachment 4.1%, persistent hyperplastic vitreous (PHPV) 8.1%, and Coats disease was 4.7% in children with white pupillary reflex [**14**].

White pupillary reflex requires the earliest attention because it can endanger not only the vision of the patient but also the life. Depending on the site of the lesion, pupillary reflex may be normal in room light but has no normal reflex on distant direct ophthalmoscopy [**15**].

Diagnosis of leukocoria requires a detailed history about the onset of symptoms, duration of white pupillary reflex, trauma, family history of retinoblastoma followed by careful clinical examination. Supporting tests and imaging include ultrasound, fluorescein angiography, optical coherence tomography, computerized tomography (CT), magnetic resonance imaging (MRI), and blood workup [**15**].

## Conclusion

In ophthalmology, leukocoria is a crucial finding that indicates an early attention and diagnosis of the underlying disease. A thorough examination is required because a delay in diagnosing the etiology can result in eyesight loss, death, or both.

**Conflict of Interest statement**

Authors state no conflict of interest.

**Informed Consent and Human and Animal Rights statement**

Informed consent has been obtained from all individuals included in this study.

**Authorization for the use of human subjects**

Ethical approval: The research related to human use complies with all the relevant national regulations, institutional policies, is in accordance with the tenets of the Helsinki Declaration, and has been approved by the review board of Fellow Pediatric Ophthalmology HMC Peshawar, Pakistan.

**Acknowledgements**

None.

**Sources of Funding**

None.

**Disclosures**

None.
